# High Yields of Hydrogen Production Induced by Meta-Substituted Dichlorophenols Biodegradation from the Green Alga *Scenedesmus obliquus*

**DOI:** 10.1371/journal.pone.0049037

**Published:** 2012-11-07

**Authors:** Aikaterini Papazi, Efthimios Andronis, Nikolaos E. Ioannidis, Nikolaos Chaniotakis, Kiriakos Kotzabasis

**Affiliations:** 1 Department of Biology, University of Crete, Voutes University Campus, Heraklion, Crete, Greece; 2 Department of Chemistry, University of Crete, Voutes University Campus, Heraklion, Crete, Greece; University of Hyderabad, India

## Abstract

Hydrogen is a highly promising energy source with important social and economic implications. The ability of green algae to produce photosynthetic hydrogen under anaerobic conditions has been known for years. However, until today the yield of production has been very low, limiting an industrial scale use. In the present paper, 73 years after the first report on H_2_-production from green algae, we present a combinational biological system where the biodegradation procedure of one meta-substituted dichlorophenol (m-dcp) is the key element for maintaining continuous and high rate H_2_-production (>100 times higher than previously reported) in chloroplasts and mitochondria of the green alga *Scenedesmus obliquus*. In particular, we report that reduced m-dcps (biodegradation intermediates) mimic endogenous electron and proton carriers in chloroplasts and mitochondria, inhibit Photosystem II (PSII) activity (and therefore O_2_ production) and enhance Photosystem I (PSI) and hydrogenase activity. In addition, we show that there are some indications for hydrogen production from sources other than chloroplasts in *Scenedesmus obliquus*. The regulation of these multistage and highly evolved redox pathways leads to high yields of hydrogen production and paves the way for an efficient application to industrial scale use, utilizing simple energy sources and one meta-substituted dichlorophenol as regulating elements.

## Introduction

Energy is one of the fundamental and vital to our survival elements in nature [1]. The energy balance adjusts all physical and chemical processes, from the simplest to the most complicated ones. Biodegradation, a series of oxidation-reduction reactions catalyzed by microorganisms, is one of them [2]. Fundamental research for the understanding of the biodegradation mechanism requires the determination of the factors involved in the energy balance of the system, which can contribute to novel ways, either for energy accumulation, or for the production of higher energy substances [2].

The utilization of energy depends on the chemical reactions involved during the biodegradation of a chemical compound, such as phenolic compounds. During the above process, the type (electron donor or acceptor) [3], the position (ortho-, meta- or para-substitution) and the number of the substituents in the phenolic ring [3], [4], [5] have a large effect on the energy requirements. The cells initially obtain the available energy needed to remove the halogen and then carry out the fission of the phenolic ring [6], [7]. Finally, the influx of energy depends on light and carbon availability, both of which are externally controlled parameters [5].

The production of energy in green algae takes place mainly in two valuable organelles, the mitochondria and the chloroplasts. Mitochondria house the mechanism that produces ATP through cytochromic and alternative respiratory electron transport chains [8], [9]. The cytochrome pathway utilizes complexes I (NADH dehydrogenase), II (succinate dehydrogenase), III (cytochrome *bc*_1_) and IV (cytochromic oxidase). The electron transfer activity of complexes I, III and IV is used to pump protons across the inner membrane, from the matrix into the intermembrane space. The resulting proton gradient drives the synthesis of ATP by complex V (ATP synthase). When electron flow through the cytochrome pathway is compromised, electrons are diverted towards the alternative oxidase, branched at the level of ubiquinone. When the alternative oxidase is exclusively used, electron flow and proton pumping are only coupled for electrons entering through complex I. Two of the three energy coupling sites are not in use and as a result the produced ATP is lower than the corresponding of the cytochromic pathway [8].

Chloroplasts house the photosynthetic apparatus that produces ATP via the linear or the cyclic electron transport chains [10] and additionally through chlororespiration [11], [12], [13] and photosynthetic hydrogen production [14], [15], [16], [17], [18]. During chlororespiration, a NADH dehydrogenase complex (NDH), showing homologies with mitochondrial complex I, transfers electrons from NADH to a quinone (presumably plastoquinone) whose reduced form would in turn be oxidized by oxygen by a plastid terminal oxidase (PTOX) [11], [12], [13]. Chlororespiration is unlikely to make a significant contribution to ATP synthesis in the light in mature chloroplasts but in immature or non-photosynthetic plastids, the contribution may become important. However, the NDH component of the chain may have a role in cyclic electron flow in the light, possibly directly or by poising the intersystem electron transfer chain in an appropriate state for cyclic electron transfer via ferredoxin-mediated pathways [19].

Photosynthetic hydrogen production by green algae was firstly reported using *Scenedesmus obliquus* in seminal experiments performed by Gaffron [14]. Light-mediated hydrogen production is attributed to a hydrogenase enzyme with high specific activity, under anoxic conditions. The photosynthetic apparatus in green algae is essential for the generation of hydrogen. The energy provided by light facilitates the oxidation of water molecules, the release of electrons and protons and the endergonic transport of these electrons to ferredoxin. Ferredoxin under anoxic conditions serves as a physiological electron donor to hydrogenase, and thus links the hydrogenase to the photosynthetic electron transport chain [14], [15], [16], [17], [18]. Electrons for hydrogen photoproduction are supplied by the photosynthetic electron transport chain, originating either from water oxidation by photosystem II (PSII) (as explained above) and/or from the metabolic oxidation of endogenous substrate in the chloroplast via its attendant electron flow to the plastoquinone (PQ) pool [20]. The highest rates of hydrogen production are typically observed in the light after anaerobic induction [15],[21]. When deprived of sulfate nutrients, the activity of PSII in *Chlamydomonas reinhardtii* declines [22] to the point where O_2_ consumption by respiration is greater than the rate of photosynthetic O_2_ evolution [23], [24]. Sealed cultures under these conditions become anaerobic in the light and produce hydrogen gas for several days.

The present contribution is a new insight in the bio-hydrogen production, since it is merging two theoretically separate topics, “biodegradation of toxic one meta-substituted dichlorophenols (m-dcps)” and “bio-hydrogen production” resulting in higher hydrogen productivities (>100 times higher than previously reported) in chloroplasts and mitochondria of the green alga *Scenedesmus obliquus*. We provide a new energy accumulating biotechnological system, which is based on controlling the energy flow by utilization of the biodegradation of m-dcps. In particular, we present that reduced m-dcps (biodegradation products) mimic endogenous electron and proton carriers in chloroplasts and mitochondria, inhibit PSII activity (and therefore O_2_ production) and enhance Photosystem I (PSI) and hydrogenase activity. This biodegradation-controlled method leads to very high yields of hydrogen production by the green alga *Scenedesmus obliquus*.

## Materials and Methods

### Organism and Growth Conditions

Cultures of the unicellular green alga *Scenedesmus obliquus*, wild type D3 [25] were grown in liquid culture medium (modified Bishop & Senger medium) [26]. The culture medium of *Scenedesmus* cultures consists of ΚΝΟ_3_ 8 10^−3^ Μ, NaCl 8 10^−3^ Μ, Νa_2_ΗPΟ_4_.2Η_2_Ο 10^−3^ Μ, ΝaΗ_2_PΟ_4_.2Η_2_Ο 3 10^−3^ Μ, CaCl_2_.2H_2_O 10^−4^ M, MgSO_4_.7H_2_O 10^−3^ Μ, Fe(III)-citrate 10^−3^ M, Fe_2_(SO_4_)_3_.7H_2_O 7.5 10^−6^ M, H_3_BO_3_ 4.5 10^−5^ M, MnCl_2_.4H_2_O 8 10^−6^ M, ZnSO_4_.7H_2_O 7 10^−7^ M, MoO_3_ 10^−7^ M and CuSO_4_.5H_2_O 3 10^−7^ M. Mother cultures were cultivated for one week, in controlled temperature (30°C) and light (150 µmol m^−2^ s^−1^) conditions. They were continuously percolated with air for CO_2_ supply and sedimentation avoidance. Subcultures with an initial concentration of 1.5 µL packed cell volume (PCV) (mL culture)^−1^ [OD_550nm_: 0.540, dry weight: 0.3 mg (mL culture)^−1^, chlorophylls: 3 µg (mL culture)^−1^, number of cells: 4.5 10^5^ (mL culture)^−1^] were distributed into 100 mL hermetically with septa closed bottles (diameter 5 cm, height 9.5 cm) and grew mixotrophically (28 10^−3^ M glucose) [26] for all the experimental procedures. The final culture volume in each bottle was 50 mL. The experiments were performed in a temperature controlled room (30°C) under a light intensity of 50–60 µmol m^−2 ^s^−1^. One meta–substituted dichlorophenol [m-dcps: 2,3-dichlorophenol (2,3-dcp), 2,5-dichlorophenol (2,5-dcp) and 3,4-dichlorophenol (3,4-dcp)] (SIGMA CHEMICAL CO, St. Louis, MO) was dissolved in methanol and added in a concentration of 0.15 mM in each hermetically closed bottle with culture [the final concentration of methanol in the cultures was 0.01% (v/v)]. The corresponding methanol amount was added also to the control cultures (absence of m-dcp). The entire m-dcp incubation time was 5 days.

The conditions of the glucose doping cultures experiments were identical to the above conditions with the exception of glucose (28 10^−3^ M) presence also in the mother cultures.

For the sulphur depletion treatments any sulphur source was removed from the treatment medium at the onset of the experiment. All other conditions were identical to the standard ones. In the culture medium of *Scenedesmus obliquus* sulphur exists in sulphate form of several salts [MgSO_4_.7H_2_O, ZnSO_4_.7H_2_O, CuSO_4_.5H_2_O and Fe_2_(SO_4_)_3_.7H_2_O]. In order to avoid further nutrient depletion (besides sulphur) the appropriate ions were supplied in the form of non-sulphur containing salts (MgCl_2_.6H_2_O 8.2 10^−4^ M, ZnCl_2_ 3.3 10^−7^ M, CuCl_2_.2H_2_O 2 10^−7^ M and FeCl_3_.6H_2_O 7.71 10^−6^ M).

Each treatment included three independent bottles and two samplings were carried out of each individual bottle using sterile syringes without opening the bottles.

### Determination of Growth

The culture’s growth rate was estimated by measuring the packed cell volume (PCV) of the culture according to the method of Senger and Brinkmann [27]. The PCV of a cell suspension was determined by centrifugation at 1500 g for 5 min using haematocrite tubes and expressed as µL PCV (mL culture)^−1^.

### Fluorescence Induction Measurements

The Handy Plant Efficiency Analyser, PEA (Hansatech Instruments, Kings’s Lynn, Norfolk, UK) was used for the fluorescence induction measurements. The maximum yield of photochemistry (F_v_/F_m_), the functional antenna size per active reaction center (ABS/RC), the dissipation energy per active reaction center (DI_o_/RC) and the density of active photosynthetic reaction centers (RC/CS_o_) were measured according to the JIP method of Strasser and Strasser [28]. This method is based on the measurement of a fast fluorescence transient with a 10 µs resolution in a time span of 40 µs to 1 s. Fluorescence was measured at 12-bit resolution and excited by three light-emitting diodes providing a saturated light intensity of 3000 µmol m^−2^ s^−1^ of red (650 nm) light. The Handy PEA data sampling operates at a maximum frequency of 100 kHz only for the first 300 µs and then the frequency decreases. This method allows the dynamic measurement of a photosynthetic sample at a given physiological state. For the fluorescence induction curves the algal cells were incubated with m-dcps or 3-(3′,4′-dichlorophenyl)-1,1-dimethylurea (DCMU) at room temperature and recorded with a saturating red light pulse of 3000 µmol m^−2^ s^−1^ after dark incubation for 5 min [29].

**Figure 1:**
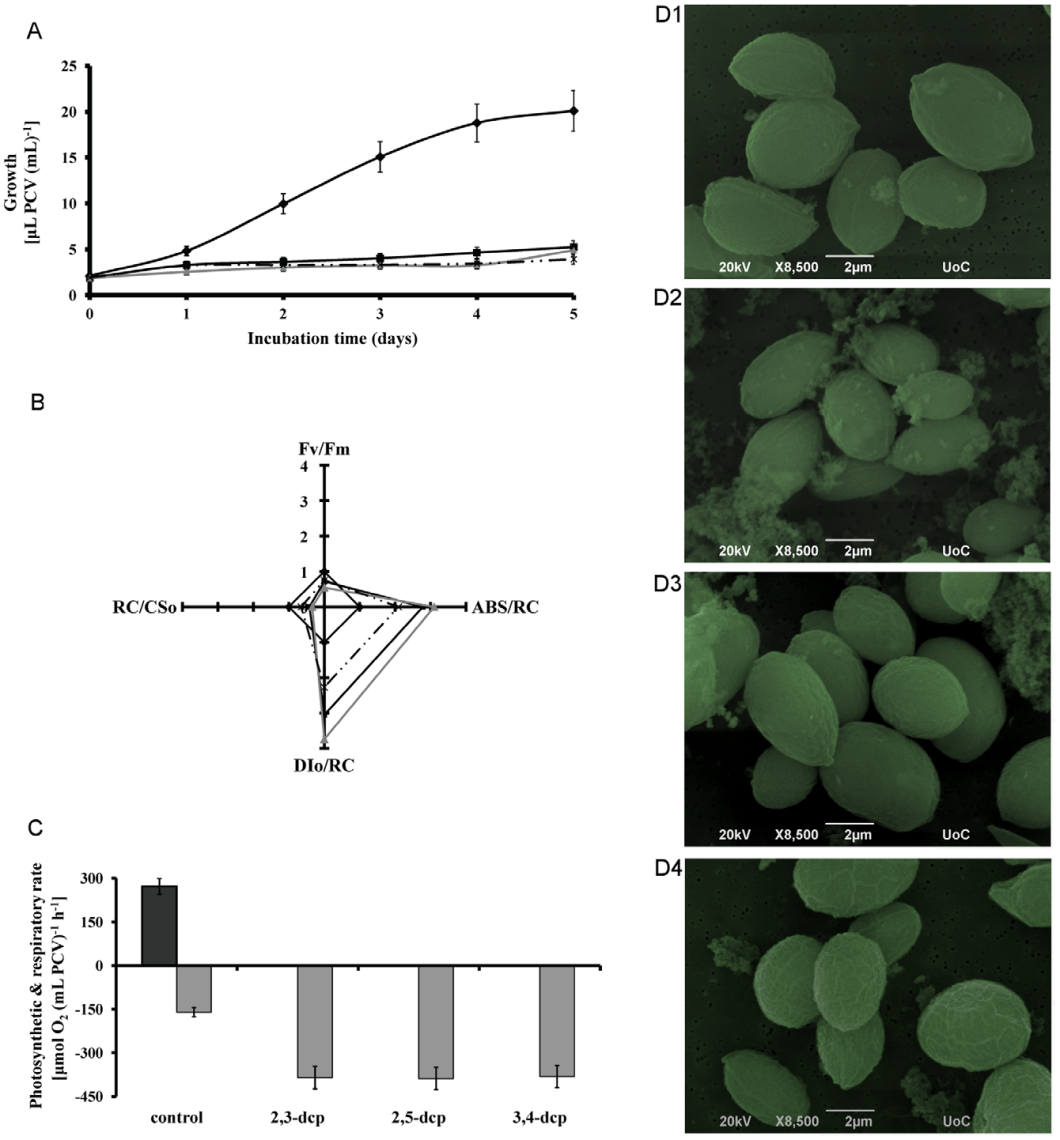
Impact of m-dcps toxicity on mixotrophic *Scenedesmus obliquus* cultures grown in the presence of 0.15 mM of m-dcps. (**A**) Growth kinetic of cultures treated with different m-dcps [in terms of µL PCV (mL culture)^−1^]. (black diamond) control, (black square) 2,3-dcp, (grey triangle) 2,5-dcp and (discontinuous line) 3,4-dcp. (**B**) Photosynthetic parameters after the first incubation day with various m-dcps [(black diamond) control, (black square) 2,3-dcp, (grey triangle) 2,5-dcp and (discontinuous line) 3,4-dcp]. F_v_/F_m_: photosynthetic efficiency (STDEV: 0.001–0.012), ABS/RC: functional antenna size (STDEV: 0.5–1.5), RC/CS_o_: density of active reactions centers (STDEV: 1.5–5), DIo/RC: dissipation energy per active reaction center (STDEV: 0.5–1.1). (**C**) Maximal photosynthetic (dark grey) and respiratory (light grey) rate of *Scenedesmus* cultures after the first incubation day with different m-dcps. (**D**) Scanning electron microscopy of *Scenedesmus* cells from cultures treated with different m-dcps. D1: control, D2∶2,3-dcp, D3∶2,5-dcp and D4∶3,4-dcp.

### High Performance Liquid Chromatography (HPLC) Analysis of Phenolic Compounds

For the phenolic compounds analysis, culture samples were centrifuged for 5 min at 1500 g and the supernatants injected into HPLC, according to the isocratic method of Lovell et al. [30]. The analyses were performed with a Shimadzu Liquid Chromatography apparatus (LC-10AD) equipped with a SPD-M10A diode array detector (Shimadzu SPD-M10A) and a narrow-bore column (C18, 2.1×150 mm, 5 µm particle size hypersil, SUPELCO). The mobile phase was methanol:water:acetic acid (50∶49:1) at a flow rate of 0.2 mL min^−1^. Detection was by absorbance at 280 nm. The quantification of the compounds was based on the absorbances of known quantities of phenolic compounds.

**Table 1:** Growth of mixotrophic *Scenedesmus* cultures incubated for five days in hermetically with septa closed bottles without [control (5 days)] and with different m-dcps.

Treatments	OD_550_ a)	DW[mg (mL)^−1^]	Number of cells(*10^5^)c)	Chlorophylls[µg (mL)^−1^]	PCV[µL (mL)^−1^]
**Control (day 0)**	0.540	0.30	4.5	3.0	1.5
**Control (day 5)**	7.250b)	4.0	60.0	26.1	20.1
**2,3dcp (day 5)**	1.900b)	1.0	16.1	0.41	5.2
**2,5dcp (day 5)**	1.770b)	0.98	14.9	0.36	4.9
**3,4dcp (day 5)**	1.400b)	0.78	11.9	0.31	3.9

a) OD was measure with a specific absorbance cuvette (10×5×45 mm).

b) OD values higher than 0.8 were measured and calculated after the appropriate sample dilution.

c) Number of cells was measured using Neubauer chamber.

Control (day 0) represents the culture start point for all the tested treatments. OD_550_: optical density of the culture at 550 nm, DW: dry weight, PCV: packed cell volume.

**Figure 2:**
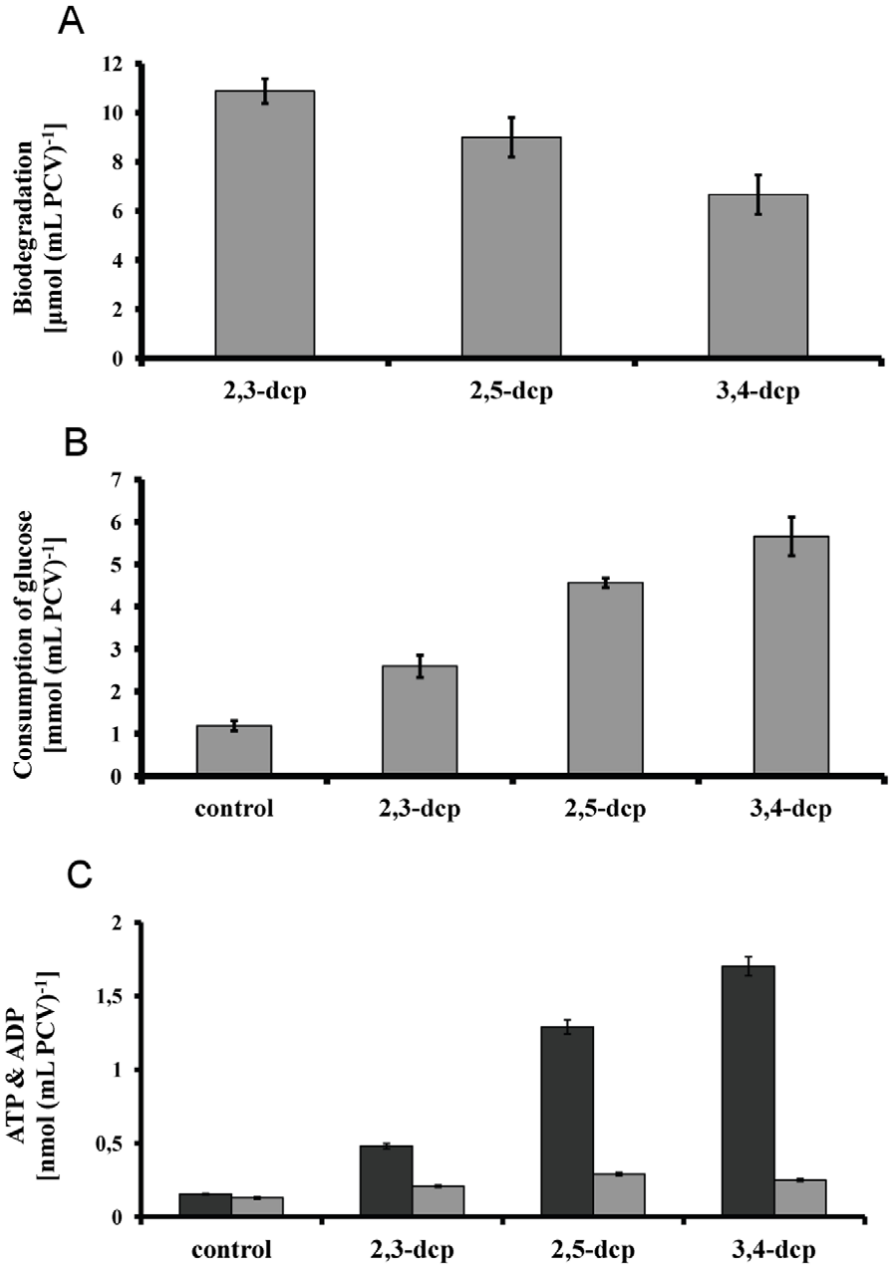
Biodegradation strategy of mixotrophic *Scenedesmus obliquus* cultures grown for five days in the presence of 0.15 mM of m-dcps. (**A**) Biodegradation of m-dcps on the fifth incubation day. (**B**) Consumption of glucose on the fifth incubation day. (**C**) ATP (dark grey) and ADP (light grey) level on the fifth incubation day.

### Polarographic Measurements

Maximal photosynthetic and respiratory rates were determined polarographically at 30°C with a Clark type electrode system (Hansatech Instruments, Kings’s Lynn, Norfolk, UK) according to the method of Delieu and Walker [31]. The actinic light (500–550 µmol m^−2^ s^−1^) was generated with a light source (MILLE LUCE M1000) and its intensity was measured with a sensitive PAR/temperature sensor (Hansatech, Quantitherm). The infrared part of the applied irradiation was filtered by inserting a 2% CuSO_4_ - containing cuvette (4 cm path length) into the light beam. The cell suspension was adjusted before each measurement to 10 µL PCV (mL culture)^−1^. This method allows the dynamic measurement of the maximal microalgal ability to produce and consume oxygen in ideal conditions (atmospheric air – no light limitation).

**Figure 3:**
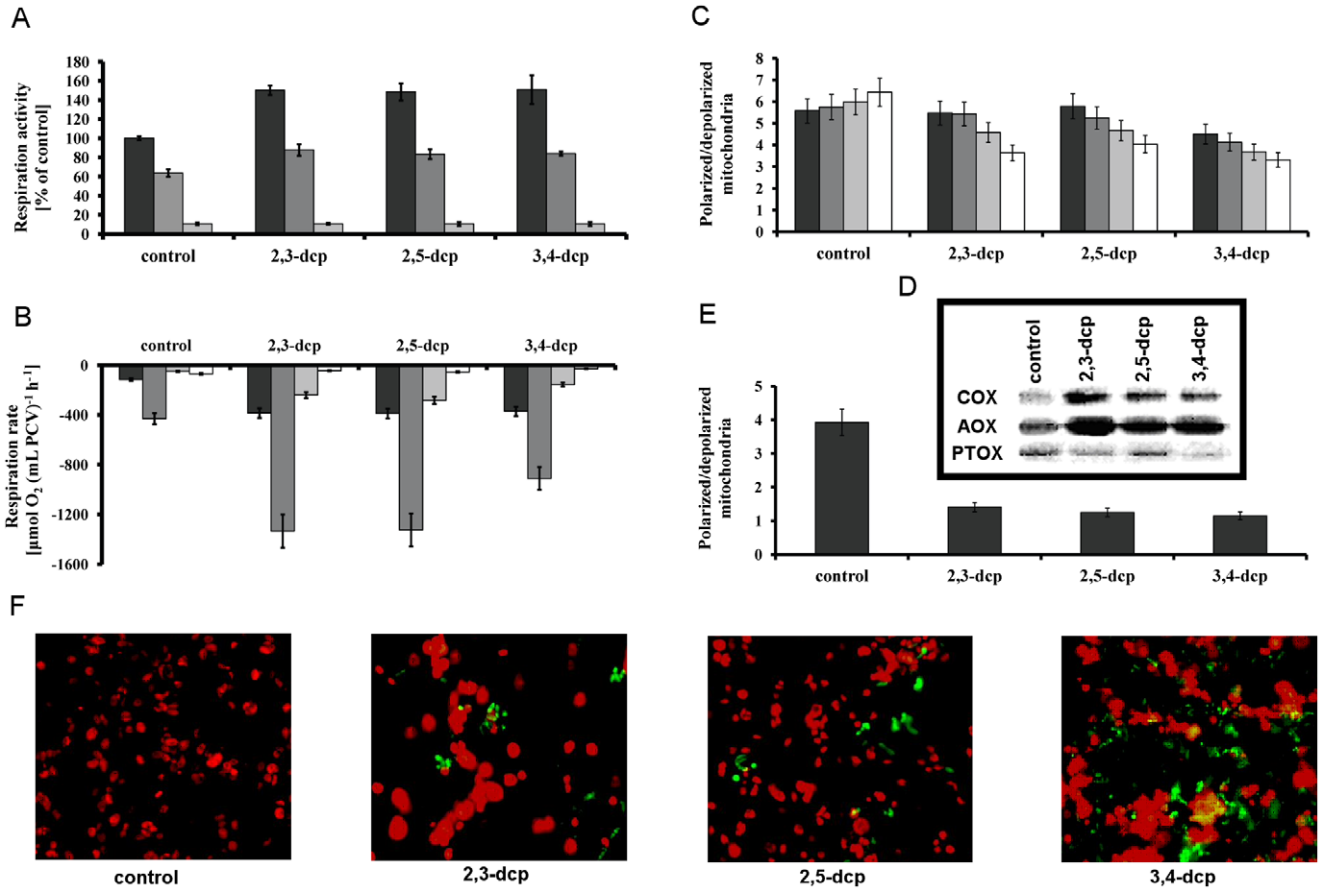
Influence of m-dcps in respiratory electron transport chains of mixotrophic *Scenedesmus obliquus* cultures. (**A**) Respiration activity in isolated mitochondria treated with m-dcps in reduced form (with ascorbate) (dark grey), rotenone + m-dcps in reduced form (grey), rotenone + antimycin + m-dcps in reduced form (light grey). (**B**) Respiration rate (measured polarographically as consumption of oxygen) in *Scenedesmus obliquus* cultures treated with m-dcps after five incubation days. (dark grey) Total respiration rate, (grey) COX capacity (SHAM and PG used), (light grey) AOX capacity (KCN and PG used) and (white) PTOX activity (KCN and SHAM used). (**C**) Short-term effect of m-dcps on mitochondrial potential, (dark grey) after 1 min incubation with m-dcp and JC-1, (grey) after 10 min incubation with m-dcp and JC-1, (light grey) after 30 min incubation with m-dcp and JC-1 and (white) after 1 h incubation with m-dcp and JC-1. (**D**) Western blot analysis of immunoreactive COX, AOX and PTOX proteins in the fifth incubation day with m-dcps (the same pattern observed in the second incubation day with m-dcps). (**E**) Long-term effect of m-dcps on mitochondrial potential after 5 days incubation with each m-dcp in the hermitical closed bottles. (**F**) Fluorescence microscopy of cells incubated with m-dcps for five days in the hermitical closed bottles.

### Measurements of COX capacity, AOX capacity and PTOX activity

Cytochrome oxidase (COX) capacity, alternative oxidase (AOX) capacity and plastid terminal oxidase (PTOX) activity were measured polarographically in the presence of the AOX inhibitor salicylhydroxamic acid (SHAM), the COX inhibitor potassium cyanide (KCN) and the inhibitor of AOX and PTOX n-propyl gallate (nPG) for a period of 10 min prior to measurements.

COX and AOX exist in the same organelle (mitochondrion), so if we block one pathway we measure the capacity of the other and not the electron flow of the blocked one [32]. Measurements for COX capacity were carried out using the inhibitors SHAM and PG, for AOX capacity using KCN and PG, while for PTOX activity using KCN and SHAM, as described by Andronis and Roubelakis-Angelakis [33]. Alternatively, PTOX activity can be measured using SHAM for AOX inhibition followed by PG insertion for estimating the residual PTOX activity, according to Cournac et al. [34], [35]. Both PTOX activity estimation procedures led to similar results.

**Figure 4:**
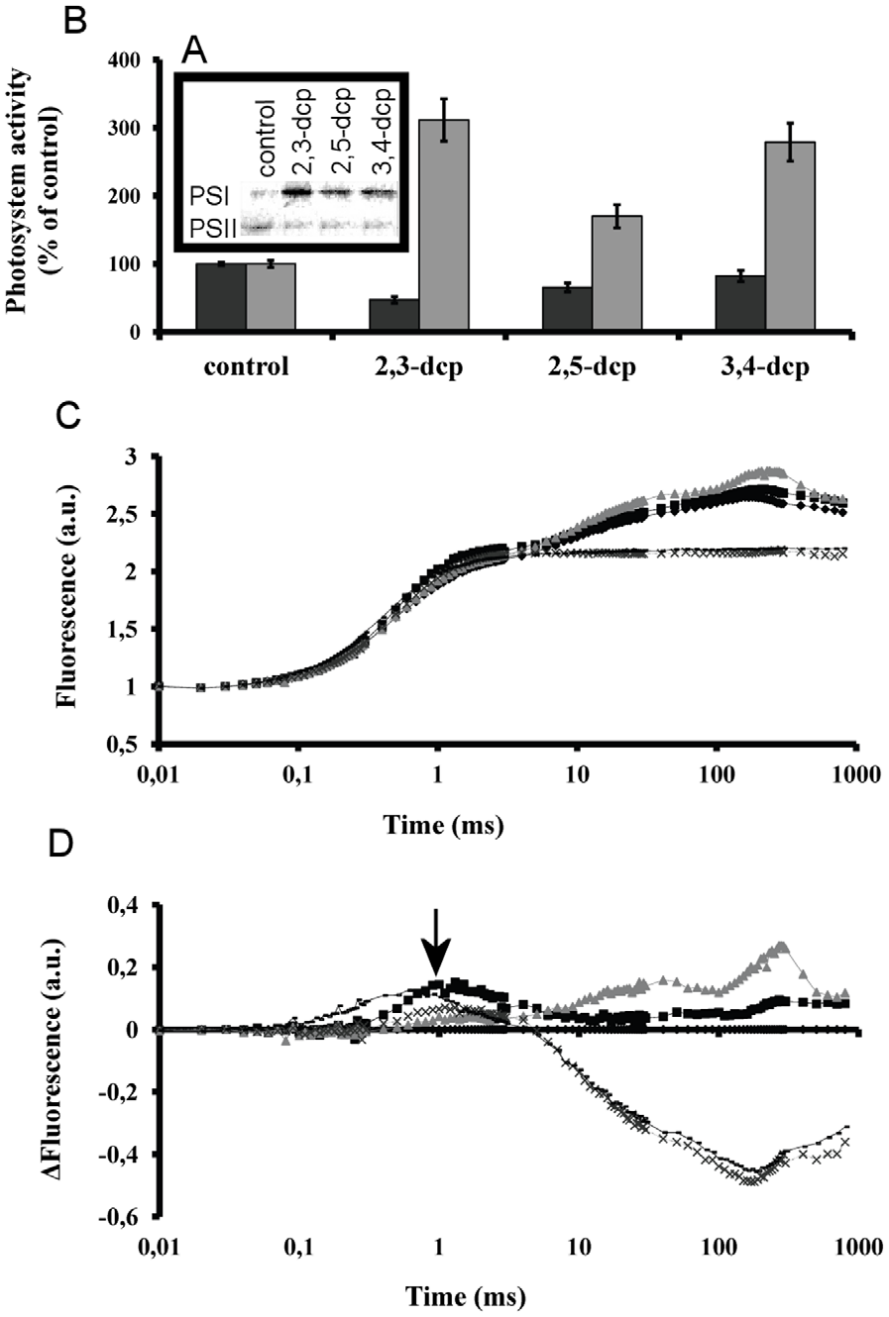
Influence of m-dcps on photosynthetic electron transport chain of mixotrophic *Scenedesmus obliquus* cultures. (**A**) Western blot analysis of immunoreactive D_1_ and PSaA proteins in the fifth incubation day with m-dcps (the same pattern observed in the second incubation day with m-dcps). (**B**) PSII (dark grey) and PSI (light grey) activities from isolated thylakoids treated with m-dcps in reduced form for 1 min. (**C**) Short term *in vivo* effects of m-dcps in primary photochemistry. Fluorescence induction curves of algal cells incubated for 5 min in the dark with m-dcps or DCMU recorded at room temperature with a saturating red light pulse of 3000 µmol m^−2 ^s^−1^. (black diamond) control, (black square) 2,3-dcp, (grey triangle) 2,5-dcp, (discontinuous line) 3,4-dcp and (continuous line) DCMU. (**D**) Difference fluorescence induction curves for the samples of Figure 4C by subtracting control values at each time point from treated samples. (black diamond) control, (black square) 2,3-dcp, (grey triangle) 2,5-dcp and (discontinuous line) 3,4-dcp and (continuous line) DCMU. The black arrow indicates the phase of inhibition of electron transport at approximately the first ms of illumination.

**Figure 5:**
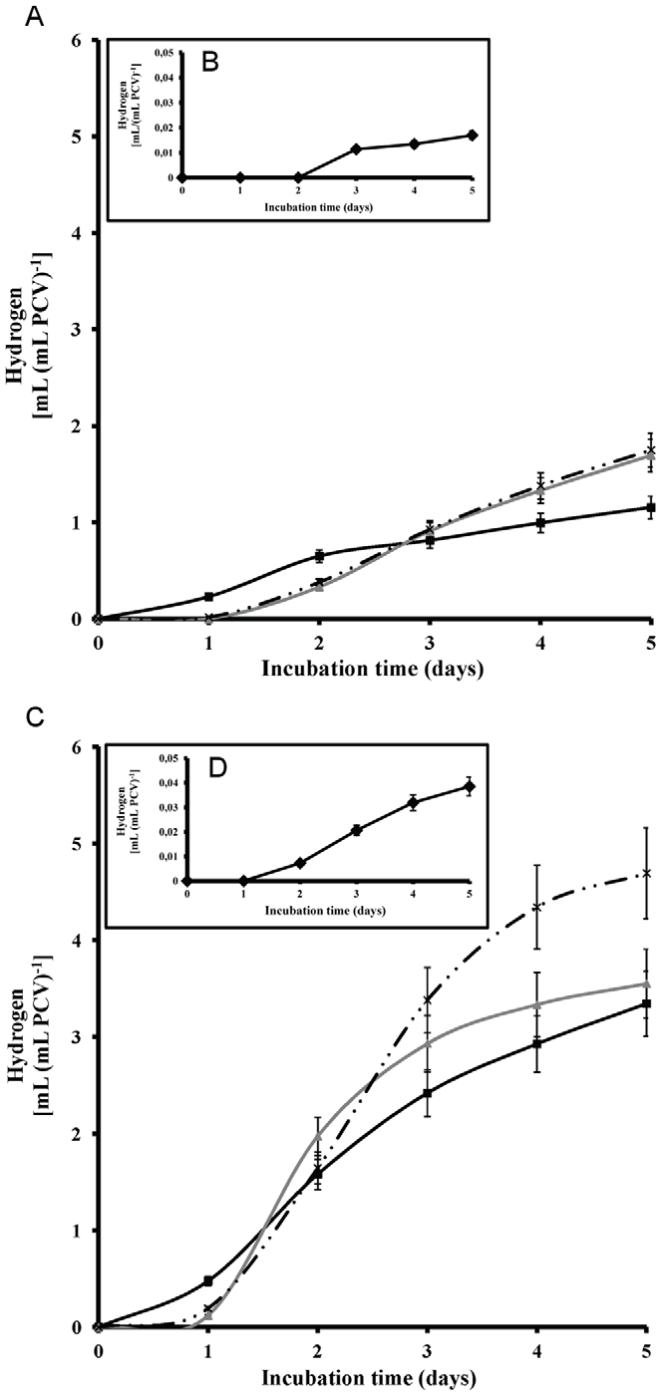
Hydrogen production measurements. (**A**) Kinetic of hydrogen production from *Scenedesmus* cultures in air-atmosphere (oxygen presence at the onset of the experiment) incubated with 2,3-dcp (black square), 2,5-dcp (grey triangle) and 3,4-dcp (discontinuous line). (**B**) Kinetic of hydrogen production for control culture in air-atmosphere. (**C**) Kinetic of hydrogen production from *Scenedesmus* cultures in N_2_-atmosphere (oxygen depletion at the onset of the experiment) incubated with 2,3-dcp (black square), 2,5-dcp (grey triangle) and 3,4-dcp (discontinuous line). (**D**) Kinetic of hydrogen production for control culture in N_2_-atmosphere.

### Isolation of Mitochondria

For the preparation of mitochondria the cultures were centrifuged for 5 min at 1500 g and the pellets were then resuspended in ice-cold mitochondria suspension buffer (20 mM MOPS-KOH pH 7.5, 300 mM sucrose, 1 mM EDTA, 1 mM MgCl_2_, and 0.2% w/v BSA) in a 1∶4 ratio. Tissue was homogenized using glass beads (ø 0.2 mm) and broken 4 times for 1 min in a cell mill (Biospec, OK, USA). The homogenate was centrifuged at 200 g for 5 min to separate the organelles (supernatant) from the rest of the cell constituents. The supernatant was centrifuged at 2000 g for 5 min to obtain a mitochondrial suspension (supernatant). The mitochondrial suspension was centrifuged at 12000 g for 10 min to pellet the mitochondria. The mitochondria pellet was resuspended in 2 mL suspension buffer, and was then purified using a percoll gradient (10, 21, 27, 45 and 60% percoll in 20 mM MOPS-KOH, pH 7.5, 0.2% BSA) at 30000 g for 30 min using a Beckman SW28 swing bucket rotor in a Beckman L8-M ultracentrifuge stopped without brake. The purified mitochondria were then washed in suspension buffer [33].

**Table 2:** Comparison of the H_2_ production from the present contribution with the corresponding ones from the literature.

References	Alga	Method of H_2_ estimation	Duration	Maximal H_2_ production	Estimated H_2_ production from the present contribution in the given literature parameters	Fold excess of H_2_ production*^e)^
Present contribution	*Scenedesmus obliquus*	Gas chromatography	5 d	*a) 4.7 mL Η_2_ (mL PCV)^−1^ *b) 12.3 mL Η_2_ (mL PCV)^−1^	**–**	**–**
Ref. [58]	*Chlamydomonas reinhardii*	Mass spectrometer	5 d	*c) 0.7 µmol H_2_ (µg chls)^−1^	1.49 µmol H_2_ (µg chls)^−1^	2–5.5 x
Ref. [16]	*Chlamydomonas reinhardii*	Mass spectrometer	2 d	12.2 nmol (µg chls h)^−1^	*d) 25.2 nmol (µg chls h)^−1^	2–5,4 x
Ref. [66]	*Chlamydomonas reinhardii* mutant *sta6*	Mass spectrometer	5 d	*c) 420 µmol (10^−9^ cells)	389 µmol (10^−9^ cells)	1–3 x
Ref. [59]	*Chlamydomonas reinhardii* mutant *Stm6*	Gas chromatography	5 d	*c) 7.7 mL Η_2_ (mg chls)^−1^	33.4 mL Η_2_ (mg chls)^−1^	4.3–11.3 x
Ref. [23]	*Chlamydomonas reinhardii*	Gas chromatography	2 d	5.94 µmol (mg chl h)^−1^	*d) 25.2 µmol (mg chls h)^−1^	4.2–11 x
Ref. [69]	*Scenedesmus obliquus*	Amperometrically	16 h	2.5 µmol H_2_ (mL PCV)^−1^	*d) 210 µmol (mL PCV)^−1^	84–220 x
Ref. [67]	*Chlamydomonas reinhardii*	Gas chromatography	5 d	*c) 35 µmol (mL gas phase)^−1^	130 µmol (mL gas phase)^−1^	3.7–9.7 x
Ref. [60]	*Chlamydomonas reinhardii*	Mass spectrometer	3 d	0.27 mmol H_2_ (mg chls)^−1^	1.23 mmol H_2_ (mg chls)^−1^	4.5–11.8 x
Ref. [61]	*Scenedesmus obliquus*	Warburg apparatus	5 h	212.2 µmol (mg chls)^−1^	*d) 582 µmol (mg chls)^−1^	2.7–7.1 x
Ref. [62]	*Chlamydomonas reinhardii*	Bio-gas Detector	1 d	24.4 nmol H_2_ (µg chl)^−1^	*d) 582 nmol (µg chls)^−1^	23.8–62.2 x
Ref. [63]	*Chlamydomonas reinhardii* mutant L159I-N230Y	Gas chromatography	285 h	166 mL H_2_ (g chl h)^−1^	*d) 565 mL H_2_ (g chl h)^−1^	3.4–8.9 x
Ref. [64]	*Chlamydomonas reinhardii*	Manometrically	4 h	7 µmol H_2_ (mg chl h)^−1^	*d) 25.2 µmol (mg chls h)^−1^	3.6–9.4 x

This comparison is not absolutely correct, because the conditions and the parameters used in the literature are totally different.

*a) : The maximal H_2_ production from Figure 5C.

*b) : The maximal H_2_ production from Fig. 8B.

*c) : The hydrogen production used for the comparison referred to the 5^th^ incubation day.

*d) : The rate was calculated from the 1^st^ to the 2^nd^ incubation day.

*e) : The first number represents the comparison of the H_2_ production from the present contribution (Figure 5C) [**4.7 mL H_2_ (mL PCV)** ^−**1**^] with the corresponding ones of other publications. The second number represents the comparison of our maximal H_2_ production (Figure 8B) [**12.3 mL H_2_ (mL PCV)** ^−**1**^] with the corresponding ones of other publications.

### Polarographic Measurements in Isolated Mitochondria

The possible position of m-dcps in the mitochondrial electron transport chain was confirmed polarographically with a Clark type electrode system (Hansatech Instruments, Kings’s Lynn, Norfolk, UK). Fresh mitochondria preparates (0.25 mg mL^−1^) were used and measured in the absence and presence of each m-dcp in oxidized or reduced form (determined by ascorbate), in the presence or absence of 100 µM rotenone (Complex I – rotenone sensitive) or/and 10 µM antimycin (Complex III – antimycin sensitive) according to the method of Heinen et al. [36].

**Figure 6:**
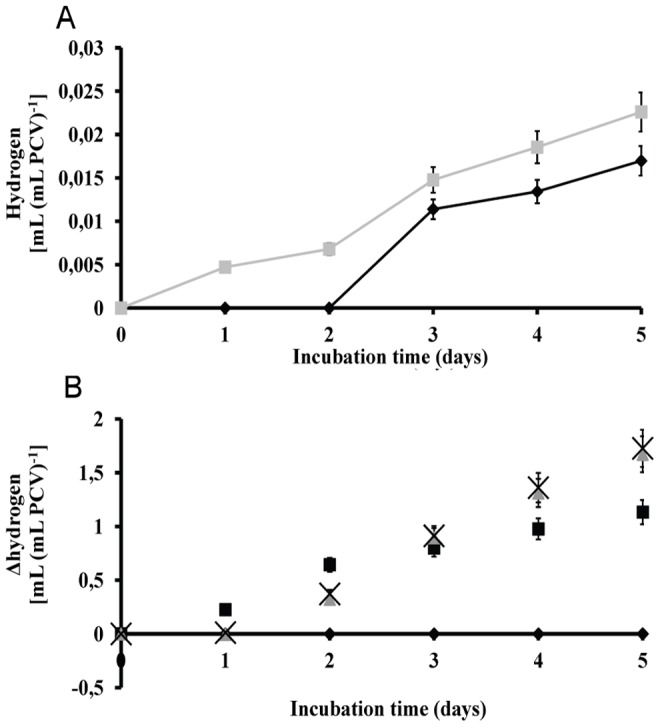
m-dcps induced H_2_ production versus sulfur depletion induced H_2_ production. (**A**) Kinetic of hydrogen production from *Scenedesmus* cultures in air-atmosphere (oxygen presence at the onset of the experiment). (black square) control and (grey triangle) sulfur depleted culture (-S). (**B**) Kinetic of Δhydrogen production from m-dcps treated cultures compared to sulfur depleted cultures (x-axe). (black diamond) -S, (black square) 2,3-dcp, (grey triangle) 2,5-dcp and (black x) 3,4-dcp.

**Figure 7:**
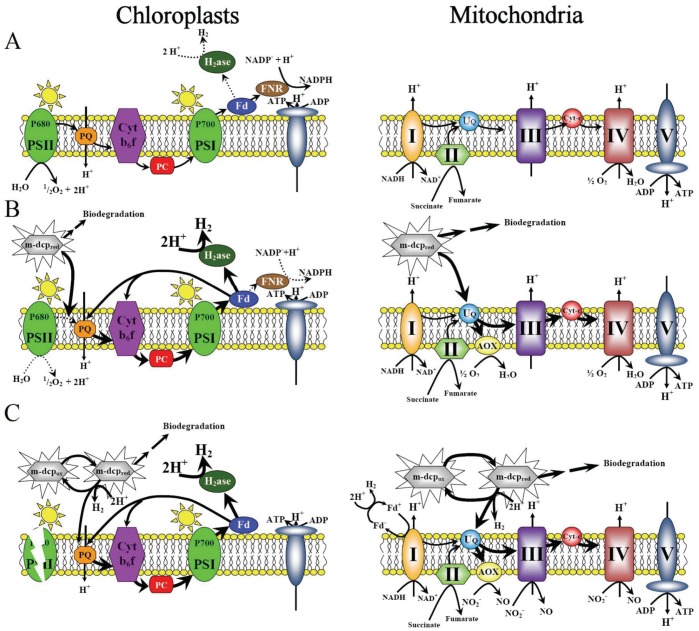
Proposed mechanisms of H_2_-production in chloroplasts and mitochondria of mixotrophic *Scenedesmus obliquus* cultures treated with m-dcps. (**A**) Aerobic conditions in chloroplasts and mitochondria before m-dcps addition. (**B**) Physiological changes in chloroplasts and mitochondria after m-dcps addition. (**C**) Oxygen depleted conditions induced by the m-dcps in chloroplasts and mitochondria.

**Figure 8:**
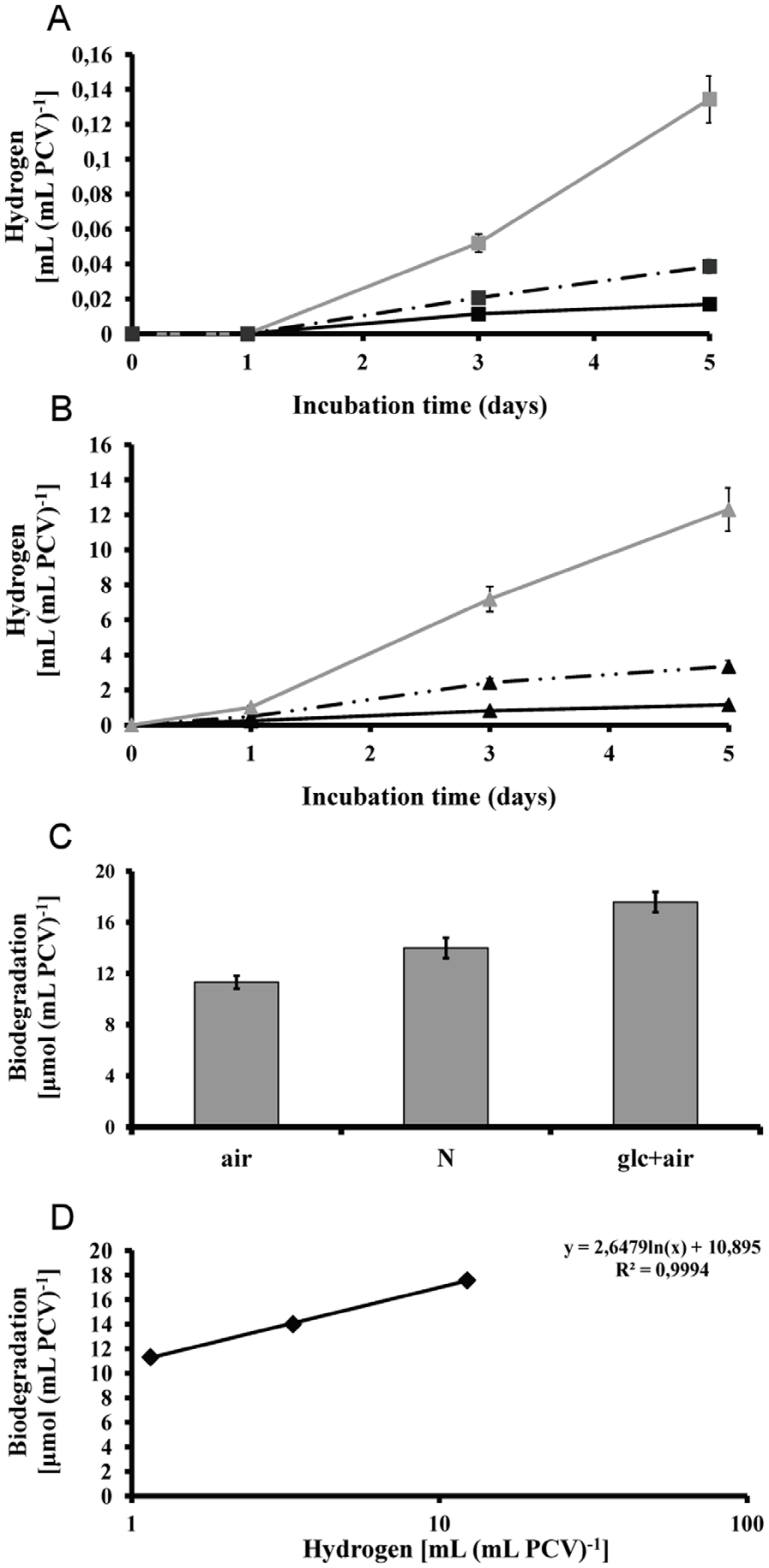
Attempts for optimization of hydrogen production. (**A**) Kinetic of hydrogen production from *Scenedesmus* cultures without any m-dcp addition. (continuous line with black square ) control in air atmosphere at the onset of experiment, (discontinuous line with black square) control in nitrogen atmosphere, (light grey square) control with additional glucose doping in air atmosphere. (**B**) Kinetic of hydrogen production from 2,3-dcp treated cultures. (black triangle) 2,3-dcp treatment in air atmosphere at the onset of experiment, (discontinuous line with black triangle) 2,3-dcp treatment in nitrogen atmosphere, (grey triangle) 2,3-dcp treatment with additional glucose doping in air atmosphere. (**C**) Biodegradation of 2,3-dcp in the above mentioned conditions. (**D**) Correlation between 2,3-dcp biodegradation and hydrogen production.

### Isolation of Thylakoids

For the preparation of thylakoid membranes the cultures were centrifuged for 5 min at 1500 g and the pellets then resuspended in 20 mM HEPES-buffer, pH 7, containing 5% glycerol (v/v). The suspension was mixed with glass beads (ø 0.2 mm) and broken 4 times for 1 min in a cell mill (Biospec, OK, USA). The homogenate was filtered through a sintered glass filter funnel to separate the glass beads, and centrifuged for 2 min at 500 g to remove unbroken cells and debris. The supernatant was centrifuged for 30 min at 13000 g. The pellet consisted of two layers. The upper green layer, enriched in thylakoid membranes, was transferred into HEPES-buffer (see above). The lower part of the precipitate contained mainly starch and was discarded [37].

### Measurement of Photosystem I and II Activities from Isolated Thylakoids

Fresh thylakoid membranes [0.15 µg Chl (mL)^−1^] were used for the determination of the activity of the photosystems, according to the method of Sudhir et al. [38]. Photosystem I (PSI) catalysed electron transport activity was assayed with DCPIP (2,6-dichlorophenol indophenol), ascorbate, DCMU [3-(3′,4′-dichlorophenol)-1,1-dimethylurea] and MV (methylviologen). The electron transport from photosystem II (PSII) to MV was measured by using DPC (diphenylcarbazid).

### Pigment Extraction and Quantitative Estimation

After centrifugation of the culture at 1500 g for 5 min, the algal pellet was exhaustively extracted with hot methanol until it was colorless. The amount of total chlorophyll was estimated photometrically according to the method of Holden [39].

### Glucose Determination

Ascensia CONTOUR strip tests were used for glucose quantification.

### ATP and ADP Extraction and Determination by HPLC

Extraction for determination of ATP and ADP was carried out according to Finazzi et al. [40]. ATP and ADP content of the extract was determined by HPLC according to Valle et al. [41].

### Measurement of Η_2_-production by GC-TCD

The hydrogen production was measured by gas chromatography, using a thermal conductivity detector (GC-TCD) (Hewlett Packard 5890 Series II). To separate hydrogen, argon was used as the carrier gas under five bars of pressure and at oven temperature of 180°C. The temperature of TCD was set at 170°C for the detector and 160°C for the injector. A gas-tight syringe (250 µL) was used for sampling from the hermetically closed bottles. The quantification of hydrogen was done by injected of known quantities of hydrogen in the GC-TCD.

### Protein Extraction and Quantification

Total proteins were extracted from cells, according to the method of Siminis et al. [42]. In brief, extraction buffer consisted of 0.2 M Tris-HCl (pH 8.0), 5 mM dithiothreitol, 0.5 mM phenylmethylsulfonyl fluoride, 10 mM leupeptin, 10% (w/v) glycerol, 0.25% (w/v) Triton X-100, and 20% (w/v) insoluble polyvinylpolypyrrolidone. The samples were homogenized with extraction buffer using a Polytron (Ultra Turrax T25, probe S15 n 10G) at a speed of 20000 rpm. The homogenates were centrifuged at 40000 g for 30 min and the supernatants divided into aliquots and frozen at −80°C. The entire extraction procedure was performed at 4°C. Protein determination was performed according to Lowry et al. [43].

### Western Blotting

Protein extracts were electrophoretically resolved using 12% SDS-PAGE, transferred to membranes and hybridised against PsaA (Agrisera), D_1_ (by D. Ghanotakis, University of Crete, Department of Chemistry), PTOX (by M. Kuntz, Laboratoire de Génétique Moléculaire des Plantes, CNRS-Université Joseph Fourier, France), AOX (by T. Elthon, MSU-DOE Plant Research Laboratory and Biochemistry Department, Michigan State University) and COX (Agrisera), according to Agrisera protocols.

### Fluorometric and Microscopic Measurements for the Polarization of Mitochondrial Membrane

Cayman’s JC-1 marker (5,5′,6,6′-tetrachloro-1,1′,3,3′,-tetraethylbenzimi-dazolylcarbocyanine iodide) was used to study the behaviour of mitochondria in the presence of m-dcps. The main advantage of this assay is that the changes in Δψ are reflected by different forms of JC-1 as either green or red fluorescence. Healthy cells with mainly JC-1 J-aggregates can be detected using a fluorophotometer (PERKIN ELMER, Luminescence Spectrometer, LS 50 B) with fluorescence settings at excitation 520–570 nm and emission 595 nm, while the monomeric (apoptotic) form of JC-1 at excitation and emission wavelengths of 485 nm and 535 nm, respectively. Long-term (incubation time 5 days) experiments were carried out following Cayman’s protocol. Short-term (1 min, 10 min, 30 min and 1 h) experiments took place differently, since m-dcp and JC-1 were added at the same time (zero time) to the tested sample. The apoptotic effect was then monitored in a time-drive process up to 1 h, as explained above. Each m-dcp was dissolved in methanol and added at a concentration of 0.15 mM to the tested with JC-1 sample. The corresponding methanol amount was also added to the control sample that was tested with JC-1 in order to exclude methanol effect.

Results from long-term experiments were examined also by fluorescence microscopy (Nikon ECLIPSE E 800), using the appropriate filter sets (B-2A: excitation 450–490, DM 505, BA 520 and G-2A: excitation 510–560, DM 575, BA 590), as explained by Cayman’s assay protocol.

### RedOx Potential Measurements

A platinum electrode was used for the RedOX potential measurements and the calculations were based on the following equation: RedOx (mV) = ± |ΔRedOx| + |ΔpH|. |ΔRedOx| is the change of the mV of the sample measured with the platinum electrode compared to the control. |ΔpH| is the change of mV of the sample measured with the argentum electrode compared to the control.

### Scanning Electron Microscopy

For scanning electron microscopy, microalgae were fixed in 2% glutaraldehyde, 2% PFA in 0.08 M sodium cacodylate buffer, pH 7.4, for 24 h at 4°C, washed in the above mentioned buffer, post-fixed in 2% aqueous OsO_4_ for 60 min at 4°C, and dehydrated through a graded series of ethanol. Dehydrated samples were critical point dried (Baltec CPD 030) and mounted on copper stubs prior to sputter coating with 20 nm thick gold/palladium (Baltec SCD 050). Samples were examined using a JEOL JSM-6390LV scanning electron microscope, operating at 20 kV.

### Data Analysis

Each treatment included three independent bottles and two samplings were carried out of each individual bottle. Standard deviations of the average values are presented on diagrams.

## Results and Discussion

It is well established that the selection of the appropriate conditions is the key for energy flow regulation and successful biodegradation. Factors such as carbon supply, light intensity, position and kind of the substitute in the phenolic ring affect the cellular energy balance and in turn the biodegradability [3], [5]. It is known that the most halogenated phenols are biodegradable if there is an adequate carbon supply [3], [5], [44], [45]. *Scenedesmus obliquus* cultures were tested for their ability to biodegrade m-dcps in the presence of light and glucose as energy sources (mixotrophic growth).

### Impact of m-dcps on the Molecular Structure and Function of the Photosynthetic Apparatus

Chlorophenols are the most energy demanding compounds compared to bromo- and iodo- phenols. The reason for this is based on the ΔΗ*_298_* values (ΔΗ_C-Cl_ = 432 kJ/mol, ΔΗ_C-Br_ = 370 kJ/mol and ΔΗ_C-I_ = 295 kJ/mol) required for the fission of one carbon-halogen bond. The presence of a second chloride (dichlorophenols) in the phenolic ring requires more energy than monochlorophenols [46], [47]. Also, meta-substitution (instead of ortho- or para- substitutions) of a chloride requires much more energy for the biodegradation of the molecules [5], [48].

The above energy data render m-dcps as quite toxic compounds. Toxicity signs appeared in the algal growth (Figure 1A). An enormous growth rate inhibition was observed in the presence of m-dcps up to 80% compared to control in the fifth day of incubation (Figure 1A and Table 1) without notably changes in the cell size and morphology, as observed in scanning electron microscopy (Figure 1D).

The observed parameters of the molecular structure and function of the photosynthetic apparatus were similar to the growth results. All m-dcps led to the inactivation of reaction centers (RC/CS_o_), the increase of the functional antenna size (ABS/RC), the enhancement of dissipation energy (DI_o_/RC) and consequently to the decrease of photosynthetic efficiency (F_v_/F_m_) (Figure 1B). The values listed in Figure 1B refer to the first day of incubation and were normalized, according to the control, which corresponds to the value of 1. The actual control values were F_v_/F_m_: 0.469, ABS/RC: 2.48, DI_o_/RC: 1.57 and RC/CS_o_: 32.27. The low value of F_v_/F_m_ was attributed to the presence of glucose [49]. After the first incubation day, these responses were so intense that photosystem II was entirely damaged in the presence of m-dcps, and therefore were perceived as non-detectable values of the JIP-test parameters.

To obtain further insight of the molecular structure and function of the photosynthetic apparatus, the maximal photosynthetic and respiratory rates were monitored at the end of the first incubation day (Figure 1C). It is clear that the values of net photosynthetic rate were zero for all m-dcps treatments, while the respiratory rates were enhanced (3-fold excess) compared to control. Polarographic measurements were expressed in terms of packed cell volume (PCV) and not in terms of chlorophylls or proteins in order to avoid an overestimation of our results. The presence of m-dcps decreased chlorophyll and protein content, so PCV estimation is considered safer. Independent of the expression of net photosynthetic and respiratory rates, the results showed the same trend: zero net photosynthesis and enhanced respiration rate in all m-dcps treatments compared to control (m-dcps absence).

### Biodegradation Strategy of Mixotrophic Microalgal Cultures in the Presence of m-dcps

Under the above conditions, biodegradation took place despite m-dcps toxicity (Figure 2A). Biodegradation tendency changed according to the chemistry of the molecule and the corresponding thermodynamic properties [46], [50], [51], in name 2,3-dichlorophenol (2,3-dcp) >2,5-dichlorophenol (2,5-dcp) >3,4-dichlorophenol (3,4-dcp) (Figure 2A).

The biodegradation data (Figure 2A) confirmed that the addition of glucose in the culture medium provided the appropriate energy levels for the removal of m-dcps, possibly through glucosidation [52]. The fact that glucose consumption increased with increasing m-dcps energy demands (Figure 2B) corroborates to the above observation. Comparison of ATP production between control cultures (absence of m-dcp) and those treated with m-dcps confirmed the above hypothesis (Figure 2C).

### Impact of m-dcps on Respiratory Electron Transport Chains of Mixotrophic Microalgal Cultures

Measurements of respiratory activity in isolated mitochondria proved that the reduced form of m-dcps enhanced respiration. This observation can be justified by the fact that m-dcps act as alternative electron acceptors and donors between complex I (rotenone sensitive) and complex III (antimycin sensitive) in the mitochondrial electron transport chain (Figure 3A). It is known that all m-dcps are mitochondrial uncouplers [53], [54]. Uncoupling properties seem to be associated with the presence of the phenolic hydroxyl that can dissociate at the membrane and play the part of a protonophore group [55]. The uncoupling properties of m-dcps in mitochondria were tested using the marker 5,5′,6,6′-tetrachloro-1,1′,3,3′,-tetraethylbenzimi-dazolylcarbocyanine iodide (JC-1) that can selectively enter into mitochondria and reversibly change color from green to red as the membrane potential increases. In healthy cells with high mitochondrial Δψ, JC-1 spontaneously forms complexes known as J-aggregates with intense red fluorescence. On the other hand, in apoptotic or unhealthy cells with low Δψ, JC-1 remains in the monomeric form, which shows only green fluorescence. Short- (Figure 3C) and long-term (Figures 3E and F) experiments, that took place in the presence of m-dcps, proved that m-dcps are strong uncouplers (>60% uncoupling compared to control). More intense uncoupling results were observed in the 3,4-dcp treatment, where the apoptotic effect appeared from the first minute (Figure 3C).

Based on the redox potential of substituted phenols [56], the most suitable position of m-dcps into the mitochondrial electron transport chain was at the ubiquinone level. It has been established that phenols are converted into quinones and ubiquinones by means of oxidation-reduction reactions. The insertion of reduced m-dcps at the level of ubiquinone overloaded the respiration chain before complex III, thus resulting to higher electron flow. In order to eliminate the excess electron charge, mitochondria activated the alternative respiration pathway [9]. As a result, an accumulation of immunoreactive alternative oxidase (AOX) protein was observed while the cytochrome oxidase (COX) doesn’t operate at full capacity (Figure 3D).

The data mentioned above combined with the zero values of photosynthetic activity after the first day of m-dcps addition (Figure 1C), suggest the generation of an oxygen depleted environment.

This observation was confirmed once more by polarographic measurements of the respiratory pathways of COX, AOX and PTOX (Figure 3B). It is important to point out that this method allows the dynamic measurement of the maximal microalgal ability to produce and consume oxygen in ideal conditions (atmospheric air – no light limitation), that could be quite different from the actual culture conditions in the hermetically closed bottles, mainly as the incubation time increased. Also, COX and AOX exist in the same organelle (mitochondrion), so if we block one pathway we measure the capacity of the other and not the electron flow of the blocked one [32]. That is the main reason of using the term capacity rather than the term activity for the polarographic measurements in intact cells (Figure 3B). Furthermore, total respiratory activity was lower than the COX capacity that according to Weger et al. [57] was attributed to salicylhydroxamic acid (SHAM–AOX inhibitor) effects on the cytochrome pathway.

In addition to the respiratory COX- and AOX- pathways in mitochondria the respiratory electron transport chain of chloroplasts (chlororespiration) was also checked. The immunoreactive PTOX protein was more abundant in control, than in m-dcps treated cultures (Figure 3D), as also observed in PTOX activities, measured polarographically (Figure 3B).

### Photosynthetic Electron Transport Chains of Mixotrophic *Scenedesmus* Cultures in the Presence of m-dcps

The changes in the molecular structure and function of the photosynthetic apparatus (Figure 1B), the zero values of photosynthetic activity (Figure 1C), the very low amounts of immunoreactive D_1_ protein (a PSII reaction center protein–Figure 4A) and the strong decrease of PSII activity (Figure 4B), confirmed that the inactivation of PSII started right at the time of m-dcps addition. While the activity of PSII decreased, the activity of PSI increased within the first few minutes after m-dcps addition, as indicated by polarographic measurements in isolated thylakoids (Figure 4B) and the substantial increase of immunoreactive PsaA protein (a PSI reaction center protein – Figure 4A).

The role of m-dcps in chloroplasts did not stop in the impact on the two photosystems. It is known that the first step of m-dcps’ biodegradation maintains m-dcps in a reduced form. The reduced m-dcps in chloroplasts seem to be incorporated at quinone level (before the plastoquinone pool – Figures 4C and 4D) and function as continuous electron donors (as long as the m-dcps biodegradation procedure takes place) to photosystem I.

### Indications of Hydrogen (H_2_) Production in Mixotrophic Cultures of *Scenedesmus* Obliquus in the Presence of m-dcps

The above-mentioned results in chloroplasts (zero net photosynthesis, PSII inactivation and PSI activation–Figure 4) and mitochondria (strong enhanced respiration rate–Figure 3) established oxygen depleted conditions. It is known that oxygen is a strong inhibitor of hydrogenase activity [18]. As a result, the oxygen depleted conditions in the tested m-dcps cultures comprised the first indication for the photosynthetic hydrogen production.

In addition, redox potential measurements (RedOx potential of m-dcps treated cultures: 2,3-dcp: −115 mV, 2,5-dcp: −109 mV and 3,4-dcp: −125 mV) established strong reductive conditions in m-dcps treated cultures and enhanced the indications for hydrogen production. The value of redox potential in control cell suspension was 230 mV, while at the beginning of the experiment was 350 mV. More negative redox potential results in a more reductive environment in the culture, leading to higher electron production. According to the above, in the reversible reaction 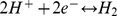, the surplus electrons lead to hydrogen production.

### Proofs for Hydrogen Production in Mixotrophic *Scenedesmus* Cultures in the Presence of m-dcps

Measurements with GC-TCD confirmed the anticipated hydrogen production. The detected hydrogen in m-dcps treated cultures was about 100-fold excess compared to control (culture without m-dcps–Figures. 5A and 5B). Hydrogen production in control cultures was so low, that it fitted in with the x-axes of the diagram A [0.018 mL H_2_ (mL PCV)^−1^ in the fifth day of incubation], so it is represented separately in the panel B. Hydrogen production is expressed in terms of PCV for two reasons. Firstly, PCV is considered safer to avoid an overestimation of our results, because the presence of m-dcps decreased chlorophylls and proteins (as explained in polarographic measurements). Secondly, it is very important to know the cell concentration, because this parameter affects total hydrogen production. More cells are expected to produce more hydrogen under the appropriate conditions.

It is worth mentioning that the order of treatments for hydrogen production was 2,3-dcp<2,5-dcp<3,4-dcp (Figure 5A), same as the order for ATP production (Figure 2C) and inverse to the biodegradation order (Figure 2A). This observation was confirmed by the polarographic measurements of PSII activity (Figure 4B). It was proven that the inactivation of PSII is much more pronounced in the case of 3,4-dcp, followed by 2,5-dcp and then 2,3-dcp. This suggests that the oxygen depleted conditions were established faster in 3,4-dcp than in 2,5-dcp and in turn faster than in 2,3-dcp.

If the establishment of oxygen depleted conditions is the main reason for the enormous hydrogen production in m-dcps cultures, then the artificial oxygen depletion at the onset of the experiment, could decrease the difference between control and m-dcps hydrogen values. Therefore, the same experiments were repeated under nitrogen atmosphere (by the onset of the experiment) to ensure oxygen depletion. Under these conditions, hydrogen production would be achieved within a shorter time, since the microalga will not waste time to create an oxygen depleted environment. Indeed, it was observed that under nitrogen rich atmosphere hydrogen production was doubled, but the trend was the same as in the non de-oxygenated cultures. Cultures treated with m-dcps showed about 125-fold excess hydrogen production compared to the anoxic control cultures (Figures 5C and 5D). Hydrogen production in the control cultures was so low, that it fitted with the x-axes of the diagram C [0.038 mL H_2_ (mL PCV)^−1^ on the fifth day of incubation], so it is represented separately in the panel D.

It is clear that m-dcps treatments under oxygenic atmosphere (at the onset of the experiment) produced about 50-fold excess hydrogen in comparison to the anoxic control (algal culture in nitrogen atmosphere without any m-dcp). This observation was clearly attributed to the presence of m-dcps and not only to the establishment of oxygen depleted conditions. It is worth mentioning that our negative control (m-dcps in culture medium without cells in the corresponding experimental conditions) did not show any hydrogen production. The above further supports the theory that biodegradation of m-dcps by the microalga is the key factor for massive hydrogen production and not the presence of m-dcps in the culture medium.

### Comparison of m-dcps Induced H_2_ Production with Previous Systems

The state of the art in hydrogen production does not help us to compare our results directly, because the majority of papers measure hydrogenase activity (which is totally different) or express hydrogen in terms of total chlorophylls or proteins or culture volume. However, in Table 2 we compare our results (after appropriate conversions to similar units) with the international literature although this comparison is not absolutely correct, because the conditions and the parameters used for hydrogen production are totally different.

Nevertheless, hydrogen production in the presence of m-dcps in terms of total chlorophylls is 2–6800 times higher [16], [23], [58], [59], [60], [61], [62], [63], [64], [65], in terms of culture density 1–3 times higher [66], in terms of gas phase 4–10 times higher [67], while in terms of culture volume 2–5 times higher [68]. Among them, only Senger and Bishop [69] express hydrogen in terms of PCV and our control value [1,7 µmol (mL PCV)^−1^] is comparable enough with their estimation [2–2.5 µmol (mL PCV)^−1^], while our best hydrogen production in the m-dcp treatments is approximately 85–220 times higher.

However, the best comparison to the literature findings comprises of the following experiment taking place in absolutely identical conditions (air atmosphere at the onset of the experiment) but in sulfur depleted cultures. Sulfur depletion is the universally known condition for hydrogen production [17], [18], [24], [70], [71], [72]. Under these circumstances *Scenedesmus* cultures without sulfur (-S) increased hydrogen production 33% compared to control (sulfur presence), but m-dcps cultures were still much higher (about 10000%) compared to control (Figures 6A and 6B). The importance of the above correlation is that the experimental conditions (light, temperature, initial cell concentration) were absolutely the same. It is obvious that the presence of m-dcps is essential for very high yields of hydrogen production.

### Proposed Working Model

In chloroplasts, m-dcps were placed in the quinone region, before the pool of plastoquinone (Figures 4C and 4D). The results were the inactivation of PSII (Figures 4A and 4B) and in parallel the activation of PSI (Figures 4A and 4B), that led to the establishment of oxygen depleted conditions (Figure 1C). During these processes, the electrons moved continuously from the reduced m-dcps through PQ and PSI to ferredoxin and then to hydrogenase where hydrogen production took place (Figure 5).

In mitochondria, m-dcps were placed in the ubiquinone region (Figure 3A) and over activated the possible routes of electron flow in the respiratory pathways that oxygen depleted conditions were installed faster (Figures 3B and 3C), contributing to continuous hydrogen production by chloroplasts.

According to the above mentioned, the oxygen depleted conditions induced in m-dcps cultures in light may help mitochondria to simply transfer the electrons onto protons, producing hydrogen, as previously reported by Embley and Martin [73]. A further possible hydrogen production route is the reduction-oxidation reactions of m-dcps (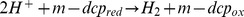) in chloroplasts and mitochondria in quinone and ubiquinone level respectively. The proposed mechanism that described the physiological changes induced by m-dcps in chloroplasts and mitochondria, which led to high yields of hydrogen production is illustrated in Figure 7.

In Figure 7A the chloroplastic and mitochondrial electron transport chains before m-dcps addition are presented. Aerobic conditions prevailed before m-dcps addition, since the consumption of oxygen by the mitochondrial respiratory pathway was lower than the photosynthetic oxygen production. Under these conditions, hydrogenase activity in chloroplasts was inhibited because the light-dependent oxidation of water in PSII released molecular O_2,_ a strong inhibitor of the enzyme.

In Figure 7B the changes induced in the above electron transport chains immediately after m-dcps addition are presented. The first step of biodegradation maintains m-dcps in a reduced form. In mitochondria the reduced m-dcps were located in ubiquinone level and overloaded the respiration chain before complex III, thus resulting to higher electron accumulation. Mitochondria activated the alternative pathway in order to get rid of excess electron charge, maximizing the total respiration rate. Concurrently, the reduced m-dcps in chloroplasts were located in quinone level (before PQ), inactivating PSII, inhibiting water photolysis, hyperactivating PSI. All these physiological changes (high O_2_-consumption without O_2_-production) established oxygen depleted conditions, which amplified the photosynthetic H_2_-production.

Lastly, Figure 7C presents the possible electron transport chains under oxygen depleted conditions. Under these circumstances, respiratory electron transport could keep on running with a different terminal electron donor, NO_2_^−^ instead of oxygen [74], [75]. In chloroplasts, the oxygen depleted conditions activated PSI, causing continuous high yields of photosynthetic H_2_-production. However, anoxia and PSI induction cannot be considered as the only reasons for the high yields of H_2_-production in m-dcps treatments. The most plausible scenario is a mechanism of mitochondrial H_2_-production, a direct H_2_-production through complex I, as described by Hrdy et al [76] and a further mechanism that came directly from m-dcps, due to their oxidation-reduction reactions (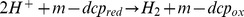) in chloroplasts and mitochondria.

The biodegradation of m-dcps is the decisive factor for the high yields of hydrogen production in green algae. The exogenously supplied glucose enhances m-dcps biodegradation (Figures 2A and 2B) and therefore the hydrogen production. A combination of these two facts in 2,3-dcp treated cultures led to approximately 12.3 mL H_2_ (mL PCV)^−1^ (Figures 8A and 8B). Among m-dcps, 2,3-dcp was selected because of its higher hydrogen production rates in primary incubation days (Figures 5A and 5C). Mixotrophic mother cultures (glucose doping) were used instead of autotrophic mother cultures in air atmosphere and then incubated again under mixotrophic conditions (as all our experiments). The effect of glucose doping appears in Figure 8A for control cultures and in Figure 8B for 2,3-dcp treated cultures. We also added the corresponding treatments of autotrophic mother cultures that were incubated in mixotrophic conditions either in air or in nitrogen atmosphere (at the onset of the experiments). The nitrogen atmosphere was added in order to show that the role of glucose is not limited to quicker establishment of oxygen depletion, but related to more bioenergetic processes, as m-dcps biodegradation. The proof of increasing biodegradation rate with hydrogen production increment is apparent in Figure 8C. The above further supports the fact that the dynamic m-dcps oxidation-reduction reactions influence the total amount of hydrogen production (Figure 8D). Further investigation of the above mentioned processes (changing the light intensity, the dose of glucose, the revictual of glucose in culture medium) can affect the total hydrogen production, but always based in the proposed mechanism and the role of m-dcps and their biodegradation.

### Conclusions

The ability of green algae to produce photosynthetic hydrogen under anaerobic conditions has been known for years. However, until today the yield of production has been very low, limiting their industrial scale use. In the present paper, 73 years after the first report on H_2_-production from green algae, we present a combinational biological system where the biodegradation procedure of one meta-substituted dichlorophenol (m-dcp) is the key element for maintaining a continuous and very high rate of H_2_-production (>100 times higher than previously reported) in chloroplasts and mitochondria of the green alga *Scenedesmus obliquus*.

In particular, we report for the first time that reduced m-dcps (biodegradation intermediates) mimic endogenous electron and proton carriers in chloroplasts and mitochondria. m-dcps inhibit photosystem II (PSII) activity (and therefore inhibit O_2_ production – which in combination with the enhanced respiration leads to oxygen depleted conditions), feed continuously electrons before the PQ-pool to Photosystem I and enhance Photosystem I (PSI) and hydrogenase activity. In addition, we show that there are strong indications for hydrogen production, from sources other than the chloroplasts, like mitochondria, in *Scenedesmus obliquus*.

The major finding of this contribution is the combination of two soundly different procedures "biodegradation of m-dcps" and "H_2_ production". The biodegradation of m-dcps is necessary for the enhanced bio-hydrogen production. Without m-dcps biodegradation the proposed H_2_ production mechanism does not work so effectively and the hydrogen productivity is more than 100 times lower. The main sources of dcps are wood pulp bleaching, water chlorination, textile dyes, oil refineries, and chemical, agrochemical and pharmaceutical industries. As a result, these toxic waste by-products could hold a regulatory role for an efficient application to industrial scale use that can produce massive hydrogen production in future, utilizing simple energy sources.
